# Converting of Myometrial Stem Cells to Tumor-Initiating Cells: Mechanism of Uterine Fibroid Development

**DOI:** 10.16966/2472-6990.e103

**Published:** 2016-04-30

**Authors:** Qiwei Yang, Michael P Diamond, Ayman Al-Hendy

**Affiliations:** Division of Translation Research, Department of Obstetrics and Gynecology, Augusta University, Medical College of Georgia, Augusta, GA, USA

Stem-cell niche is composed of a group of cells within the specific anatomic location that function to maintain stem cells. The niche referring to a microenvironment is capable of generating extrinsic factors that modulate stem cell proliferation and fate determination [1]. During development, various niche factors act on stem cells to alter gene expression, and induce their proliferation or differentiation for the development of the fetus. The highly plastic state of the stem/progenitor cells during developmental and tissue maintenance permits the required flexibility for proper tissue formation and repair. Unfortunately, this plasticity also provides an opportunity for aberrant cellular reprogramming via epigenetic mechanisms due to inappropriate exposures to toxins [2]. The developmental adverse exposure can lead to persistent, life-long effects and resulting in a variety of diseases [3].

Uterine Fibroids (UFs) are monoclonal tumors arising from the myometrium. An increasing body of evidence supports the hypothesis that UFs originate from stem cells in the myometrium, although the specific cell of origin for these tumors has remained elusive [4, 5]. The existence of stem cells from myometrium and UFs have been identified, and several studies have been performed to identify tumor-initiating cells in UFs [6–9]. Notably, the difference between myometrial stem cells (MSCs) and fibroid stem cells at DNA level is that *MED12* mutations were found only in fibroid stem cells, but not MSCs [9]. The *MED12* mutations occur in human UF tissues with high frequency in contrast to the findings that other gene mutation and genetic abnormalities occur at relatively low levels in UFs [10, 11]. Recent study demonstrated that *MED12* mutation is a driver for promoting development of UFs and genomic instability [12]. Distinct *MED12* mutations have been detected in different fibroid lesions in the same uterus [13] suggesting that the emergence of each *MED12* mutation is an independent event in altered MSCs.

Endocrine disruptors (EDs) are naturally occurring or man-made compounds that may interfere with the endocrine system and cause unfavorable developmental and reproductive effects on human. Increasing studies show that endocrine disruptors may pose the serious risk of many diseases during development [14, 15]. A number of studies demonstrate that estrogen clearly influences the proliferation and differentiation of various stem cell types. Epidemiological and experimental studies show that EDs increase the risk of tumorigenesis, especially in the organs that are extremely sensitive to endocrine regulation. In the Eker rat fibroid model, developmental exposures to EDs such as diethylstilbestrol and genistein during a crucial period of uteri development increase the penetrance and growth of UFs concomitantly reprogramming estrogen-responsive gene expression [16–18].

The adverse effect of early life exposure may cause the deregulation of multiple developmental processes including disruption of stem cell niche, developmental reprogramming and altered stem cell characteristics. The somatic stem/progenitor cells from varied tissues and organs have been shown to remain susceptible to EDs [19]. One of the important studies by Bulun’s group showed that the differentiated myometrial cells in response to estrogen and progesterone treatment resulted in secretion of wingless-type (WNT) ligands, which induced nuclear translocation of β-catenin in stem/progenitor cells from UFs. The activation of β-catenin pathway ultimately enhanced the cell growth and proliferation of these stem/ progenitor cells [20].

Taken together, emerging studies suggest that the developmental exposure to EDs and other toxins may result in genetic/epigenetic alterations and aberrant niche of MSCs, thereby converting the MSCs to tumor-initiating cells via a variety of signaling pathways. Thus further understanding of contributions of the stem cell micro-environment/ reprogramming to development of UFs will be important for future clinical progress.
